# The spectrum and burden of in-patient paediatric musculoskeletal diseases in Northern Tanzania

**DOI:** 10.1080/20469047.2022.2062561

**Published:** 2022-04-22

**Authors:** Rebecca B. Walsh, Anthony Mwingwa, Nateiya M. Yongolo, Sanjura M. Biswaro, Manasseh Joel Mwanswila, Clive Kelly, Blandina T. Mmbaga, Faith Mosha, William K. Gray, Emma McIntosh, Richard W. Walker

**Affiliations:** a Institute of Ageing and Health, Newcastle University, Newcastle upon Tyne, UK; b Department of Microbiology and Immunology, Kilimanjaro Christian Medical University College, Moshi, Tanzania; c Department of Research, Kilimanjaro Clinical Research Institute, Kilimanjaro Christian Medical Centre, Moshi, Tanzania; d Department of Internal Medicine, Kilimanjaro Christian Medical University College, Moshi, Tanzania; e Department of Health Management Systems, Kilimanjaro Christian Medical University College, Moshi, Tanzania; f Department of Paediatrics and Child Health, Kilimanjaro Christian Medical University College, Moshi, Tanzania; g Health Economics and Health Technology Assessment, Institute of Health and Wellbeing, University of Glasgow, Glasgow, UK

**Keywords:** Paediatric musculoskeletal disease, sub-Saharan Africa, inpatient admissions

## Abstract

**Background:**

Musculoskeletal diseases (MSD) are a major contributor to the global burden of disease and disability, and disproportionally affect low- and middle-income countries; however, there is a dearth of epidemiological data. Affected children often face increased morbidity, social isolation and economic hardship.

**Aim:**

To assess the spectrum and burden of paediatric MSD in children aged 5–18 years admitted to a major referral hospital in Tanzania.

**Methods:**

This was a retrospective cohort study of children aged 5–18 years admitted to Kilimanjaro Christian Medical Centre (KCMC) whose initial diagnosis was recognised as a musculoskeletal condition by the International Classification of Diseases-10 between 1 January and 31 December 2017.

**Results:**

During 2017, 163 cases of confirmed paediatric MSD were admitted to KCMC, representing 21.2% of all admissions of children aged 5–18 years (*n = *769). Bone disease was the most common diagnosis. They comprised 106 (65.0%) traumatic fractures, 31 (19.0%) osteo-articular infections, 9 (5.5%) malunions and 3 (1.8%) pathological fractures. Congenital defects and rheumatic disease were relatively uncommon, accounting for only 6 (3.7%) and 4 (2.5%) MSD admissions, respectively.

**Conclusion:**

The majority of cases of MSD were related to fractures, followed by osteo-articular infections, while recognised cases of rheumatic disease were rare. The study, although small, identified the sizeable burden and spectrum of paediatric MSD admitted to a hospital in Tanzania over a 12-month period and highlights the need for larger studies to inform the optimal allocation of health resources.

**Abbreviation:**

CI: confidence interval; HIC: high-income countries; HIV: human immunodeficiency virus; ICD-10: International Classification of Diseases 10; IQR: interquartile range; JIA: juvenile idiopathic arthritis; KCMC: Kilimanjaro Christian Medical Centre; LMIC: low- and middle-income countries; MSD: musculoskeletal diseases: NAI: non-accidental injury; NIHR: National Institute for Health Research; PAFLAR: Paediatric Society of the African League Against Rheumatism; RTA: road traffic accidents; SCD: sickle cell disease; SLE: systemic lupus erythematosus; SSA: sub-Saharan Africa.

## Introduction

Musculoskeletal disease (MSD) encompasses over 150 conditions affecting bones, joints, muscles, tendons and ligaments [1]. They are a diverse group of conditions with a wide spectrum of disease pathology and constitute a large proportion of the global burden of disease and disability [2]. According to the World Health Organization, MSD is the second leading cause of years lived with disability and the largest contributor to persistent pain globally [2,3]. However, data relating to the spectrum and burden of paediatric MSD globally are limited, with few studies from low- and middle-income countries (LMIC), of which only a handful are specific to East Africa, although the burden of disease is thought to disproportionally affect LMIC [4,5]

Although the global burden of paediatric MSD is not well defined, the impact on the individual has been described in detail. The adverse implications are not limited to the child’s physical health, which in turn increases the individual’s susceptibility to other non-communicable disease, but mental, social, and economic domains are also negatively affected [6,7]. A study investigating the effect of disability on education in LMIC reported that up to 85% of disabled primary school-age children have never attended school [8]. Access to education has been shown to be a crucial positive predictive factor for improved long-term health and wealth [6,9]. Children with paediatric MSD are not only disadvantaged in their youth but the negative effects persist into adulthood.

The lack of data from LMIC means that the burden of disease is probably under-estimated which may result in inadequate service provision. This paucity of data needs to be urgently addressed as current trends suggest that the prevalence of MSD is set to increase, reflecting current epidemiological transitions in LMIC [10]. Paediatric MSD not only puts considerable stress on an already resource-limited health service in the acute setting, but the poor prognosis and chronicity of many of these conditions can also result in lifelong health demands. Without an understanding of the burden and spectrum of disease resulting from paediatric MSD in Tanzania, the provision of adequate clinical services poses a major challenge.

Previous population-based studies in East Africa have shown that a substantial proportion of the paediatric population suffers from paediatric MSD [4,5]. However, there are few data describing the burden and spectrum of paediatric MSD in Tanzania and none related to inpatient care. This study aimed to address this by undertaking a retrospective cohort study, collecting inpatient data for 2017 from a large referral hospital in Northern Tanzania.

## Methods

### Setting

Data were collected at Kilimanjaro Christian Medical Centre (KCMC), which is one of the four largest tertiary referral hospitals in Tanzania. Situated in Moshi, Northern Tanzania, it serves a potential catchment population of 15 million people, 50% of whom are thought to be under 18 years. Of the 630 inpatient beds available at KCMC, 66 are dedicated to children, though paediatric admissions are not limited to these beds.

### Participants

Children aged 5–18 years admitted to KCMC in 2017 with a diagnosis recognised as a musculoskeletal condition by the International Classification of Diseases-10 (ICD-10) were included [11]. Children under 5 were excluded as MSD was likely to be rare and to represent a different disease spectrum.

### Study design

This was a retrospective cohort study of all children aged 5–18 years admitted to Paediatric Ward 1, Paediatric Ward 2 and the orthopaedic and dermatology wards at KCMC between 1 January and 31 December 2017. These wards were included to accurately reflect the admission pathway of children presenting at KCMC with paediatric MSD.

Data were collected by RW and AM. Phase one involved recording all admissions from the ward admission book to identify suspected cases of MSD. All admissions were recorded in the ward admission book by the sister in charge on that day.

In phase two, the medical notes of those identified in phase one as possible MSD were retrieved. A proforma was used to confirm the diagnosis and to collect basic demographic data as well as information on treatment, initial investigation findings and length of stay.

### Statistical methods and data analysis

Microsoft Excel and the Statistical Package for Social Science version 24 for Windows were used for analysis. Statistical significance was *p*<0.05. Normally distributed data were summarised using mean and standard deviation. Non-normally distributed, interval/ratio data and ordinal data were described using the mean and interquartile range (IQR).

Categorical data were summarised using frequency, and inference was drawn using the χ^2^ test. To investigate the differences in incidence rates, 95% confidence intervals (95% CI) were used, calculated on the basis of the assumptions of the Poisson distribution.

### Ethics

Ethical approval was granted locally by KCMC, Moshi, and nationally by the National Institute for Medical Research, Tanzania.

## Results

**Figure 1.**
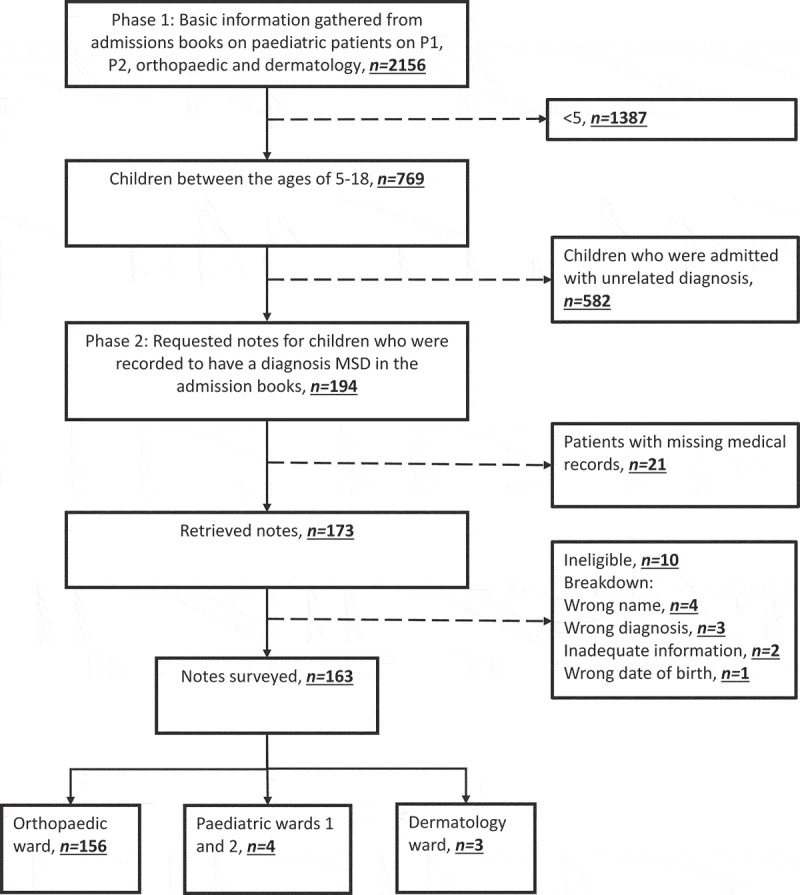
Identification of the study population.

In 2017, there were 2156 paediatric admissions to KCMC, 769 (36%) of whom were aged 5–18 years. Figure 1 outlines identification of the study population. A review of those 769 identified 194 (25.2%) with a potential diagnosis of MSD, and medical records were complete for 163 (84.0%) of them. In descending order, there were 106 (65.0%) fractures, 31 (19.0%) osteo-articular infections in the form of osteomyelitis and septic arthritis, 9 (5.5%) malunions (a fracture that has healed incorrectly in a suboptimal position), 6 (3.7%) congenital defects, 4 (2.5%) rheumatic diseases and 3 (1.8%) cases of pathological fractures.

Of the 31 identified in the ward admissions books as possible MSD for whom notes were unavailable, 20 (65%) were male and the median age was 13 years (IQR 11–15). Tribe and religion was recorded for only two children. The likely diagnosis was recorded as 24 (77.4%) fractures, 4 (12.9%) pathological fractures, 2 (6.5%) spinal cord injuries and one (3.2%) osteo-articular infection. This is broadly similar to the spectrum and burden in the confirmed cases.

Of the 163 confirmed cases, 118 (72%) were male and the median age of admissions was 11 years (IQR 8–15). Of over 120 tribes in Tanzania, 36 were represented in this cohort. Of the 159 for whom a tribe was recorded, the largest representation was as follows, 64 (39%) were Chagga, 32 (20%) Pare, 16 (10%) Maasai and 8 (5%) from the Sukuma tribe. Most children (117, 72%), were Christian, 31 (19%) were Muslim, 4 (3%) were pagan and no religion was recorded for 11 (7%).

**Figure 2.**
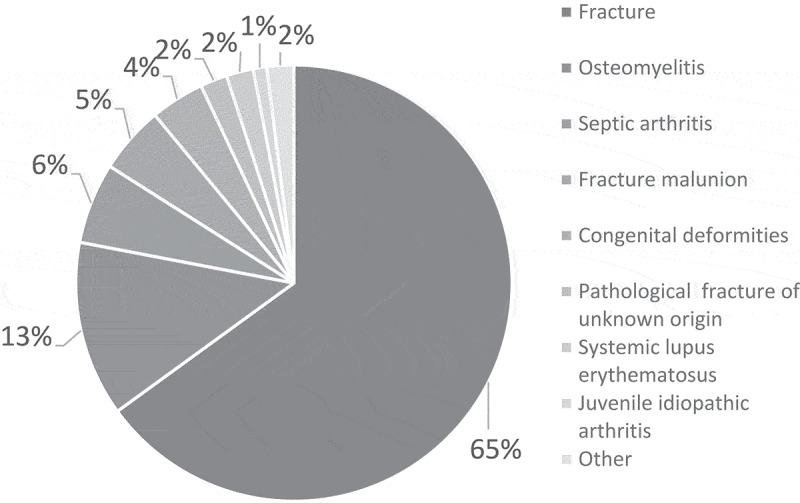
Spectrum and frequency of paediatric MSD.

The spectrum and frequency of confirmed cases of paediatric MSD are shown in Figure 2.

**Figure 3.**
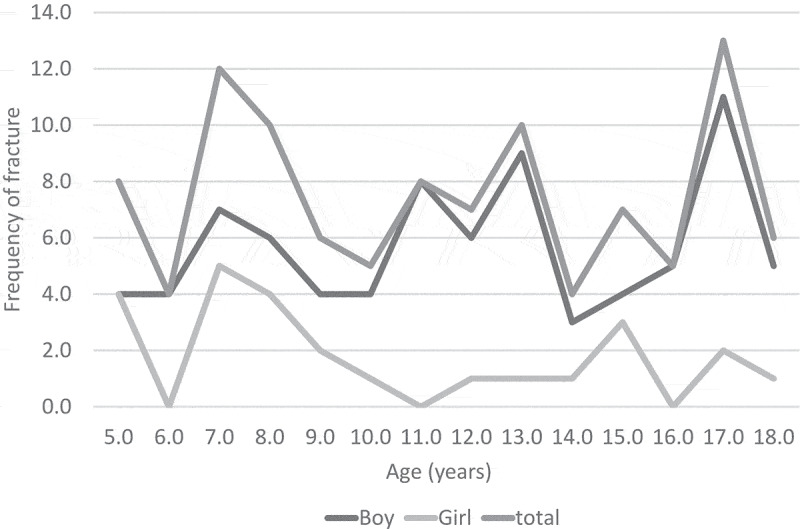
Frequency of fracture by age and gender.

At every age, the frequency of fractures was greater in males than in females (Figure 3). Of all the fractures, 81 (76.4%) were in males and 25 (23.7%) in females (χ^2^ = 3.766, *p*=0.05).

**Figure 4.**
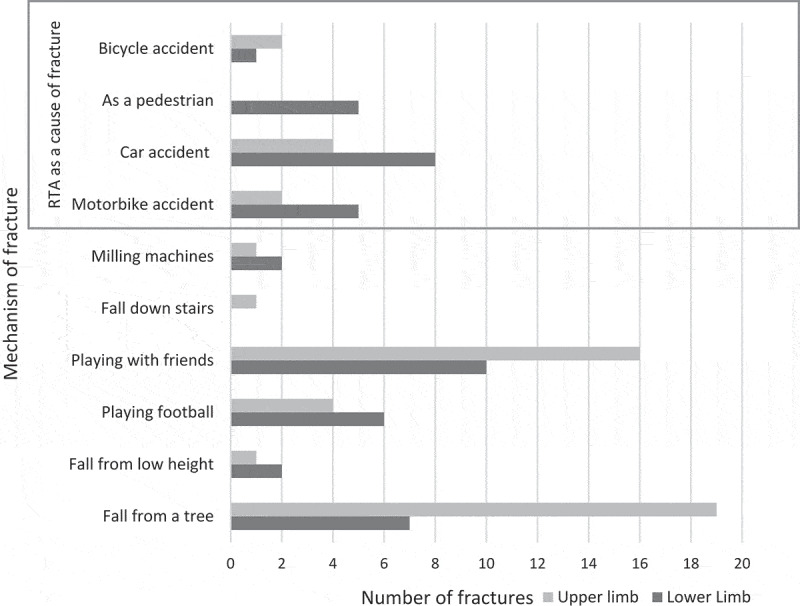
Limb fractures by mechanism of injury.

In males, 42 (51.9%) fractures involved the upper limbs, 37 (45.7%) affected the lower limbs and 2 (2.5%) had several limb fractures; in females, 12 (48.0%) fractures affected the upper limbs, 12 (48.0%) affected the lower limbs and one (4.0%) had several limb fractures. Of the 106 confirmed fractures, the cause of the injury was recorded for 96 (Figure 4).

Osteo-articular infections were the second most common paediatric MSD. There were 21 cases of osteomyelitis and 10 cases of septic arthritis. Both osteomyelitis and septic arthritis were more common in males than in females — 1:0.43 and 1:0.75, respectively. This difference is not statistically significant (osteomyelitis χ^2^ = 3.549, *p*=0.09; septic arthritis χ^2^ = 0.17, *p*=0.57). All infections involved the lower limbs.

## Discussion

This retrospective study of paediatric admissions to KCMC in 2017 highlights the broad spectrum and burden which paediatric MSD places on this health system, accounting for 21.2% of all paediatric admissions aged 5–18 years. Fractures accounted for the largest proportion of these admissions, followed by osteo-articular infections. There were small numbers of fracture malunions and pathological fractures. Rheumatic disease and congenital deformities were uncommon.

A community-based study in Uganda also reported a broad spectrum and burden of paediatric MSD, although the spectrum there was different from that in this study [5]. It identified a high prevalence of gluteal and quadriceps contractures as well as post-injection paralysis, accounting for 29.4% and 12.7%, respectively [5]. Contractures are caused by fibrosis in the muscle and/or fascia which results in reduced limb movement and deformity. While contractures can be congenital and idiopathic in nature, the most common cause is acquired secondary to intramuscular injection [12]. Repeated injection can cause ischaemia, neuropathy and the formation of haematomas [13]. The prevalences of fractures and osteo-articular infections were 5.2% and 5.3%, respectively. The difference in the spectrum of disease can be partly explained by the different study setting, community versus inpatient, and the higher number of malaria cases in the Uganda site compared with this area of Tanzania [14]. In Uganda, intramuscular injections of antimalarials are used regularly to treat malaria, resulting in a significant incidence of sciatic nerve damage and muscular fibrosis, ultimately leading to the high prevalence of paralysis and contractures seen in the Ugandan cohort [15].

In accordance with the literature, fractures were the most common paediatric MSD in this study. A study in Malawi found that 25% of all injuries in children presenting to accident and emergency were owing to fractured bones [16]. Fractures are a common occurrence in the paediatric population worldwide, reflecting the behaviour and activity common in childhood and their lower bone mass density and skeletal immaturity [17]. The predominance of fractures in males in this study also mirrors the gender pattern of fractures globally. This global trend is thought to reflect the fact that boys partake in more sport and riskier behaviour, and that there is less supervision of boys [18,19].

In this study, the ratio of lower limb to upper limb fractures was 1:1.1, considerably higher than in studies in similar settings in high-income countries (HIC) where the ratio of lower to upper limb fracture in noticeably lower 1:2.5 [20]. Lower limb fractures present a greater burden of disease to the individual as well as to the health services than upper limb fractures. Lower limb fractures require more intervention and inpatient treatment at initial presentation, such as traction and surgical correction, and they are associated with a higher rate of chronic disability which further exacerbates the health burden caused by paediatric MSD in LMIC [21].

In this study cohort, there may be an element of selection bias with lower limb fractures being disproportionally represented. A study in Ghana investigated the use and role of traditional bonesetters and found that they were often favoured over hospital treatment when the fracture was less severe [22]. With one study suggesting that over 55% of Tanzanians use traditional healers, it can be inferred that, because upper limb fractures are classically less severe, this study has under-estimated their true frequency [23]. However, the study in Ghana did not stratify fractures based on the limb affected, and the most important factor which influenced the decision whether to go to hospital was the expense, with hospital treatment costing up to twenty times more than traditional bonesetters.

Although it cannot be inferred from this study why the rates of lower limb fractures are higher in Tanzania, it is known that LMIC are disproportionally affected by road traffic accidents (RTA) and that lower limbs are the most common site of injury following an RTA [24]. This study did show that, of the fractures secondary to an RTA, 19 (70%) were lower limb and only 6 (30%) were upper limb. It has been suggested that the high incidence of RTA in LMIC might partly reflect the impact of the current uncontrolled urbanisation and rapid motorisation in such countries. This epidemiological shift is not currently being met by adequate enforcement of road and vehicle safety legislation and education [25]. Studies which investigate the mechanisms of preventable injuries would provide data to support a multi-sectoral approach to reduce preventable injuries and thus the burden of disease. In HIC, such approaches have been successful in reducing death and serious injury following RTAs [26].

Of the recorded mechanisms of fracture in this cohort, there was no non-accidental injury (NAI). Child abuse is common throughout Africa; 60% and 51% of boys and girls, respectively, experience some form of physical abuse during childhood [27]. Of those affected, up to 55% of young children will suffer a fracture. Rib fractures and spiral and oblique fractures of long bones are the most common caused by abuse [28,29]. The lack of NAI fractures in this cohort might be explained by the age group (approximately 80% of fractures caused by abuse are in children <18 months) and the well recognised problem in Africa of the under-reporting of child abuse [29,30]. When it comes to reporting abusers, victims face a multitude of barriers and deterrents which often include victim shame, stigma, distrust of authority and cultural beliefs which can cause the victim to be disowned [30].

In this study, osteo-articular infections were the second leading contributor to the burden of paediatric MSD, accounting for 19% of admissions. They present a very real threat to children’s health and wellbeing in LMIC, unlike in HIC where osteo-articular infection is becoming an issue of the past [31]. These results also support previous reports from sub-Saharan Africa (SSA) which, although published some time ago, describe the large surgical burden which osteomyelitis placed on hospitals in Uganda. Operations to treat osteomyelitis represented 3.5% of all surgical procedures, 34% of which involved children aged 10–14 years [32].

In SSA, it has been shown that most cases of osteo-articular infection result from predisposing factors such as wounds, open fractures and poorly treated infections which are common in LMIC [33]. Lower standards of living owing to deprivation can lead to poor skin hygiene, malnourishment and anaemia, all of which predispose children to infection [34]. These risk factors coupled with the higher prevalence of sickle cell disease (SCD), tuberculosis and human immunodeficiency virus (HIV) result in children in SSA being at a substantially increased risk of developing osteo-articular infection [35]. SCD predisposes individuals to salmonella infections which cause severe and invasive osteo-articular disease [36]. Bone disease is the most common cause of hospitalisation in SCD. This is predominantly owing to painful vaso-occlusive crises or infection. Other complications include avascular necrosis, stress fractures, arthritis and skeletal growth disturbance [37]. The immune-compromised state related to HIV increases children’s susceptibility to osteo-articular and tuberculous infections. Half of extra-pulmonary manifestations of tuberculosis involve the musculoskeletal system, adding further to the already sizeable burden caused by osteo-articular infections [38]. The management of these conditions is compromised by the resource-limited health and social care services common to SSA. A study that aimed to characterise the availability of MSD health services found that there were deficiencies across every level of orthopaedic provision in all the LMIC assessed [39].

In this cohort, rheumatic diseases were rare, with one admission with juvenile idiopathic arthritis (JIA) and three with systemic lupus erythematosus (SLE). A similar study in Kenya found no cases of JIA and one case of SLE [4]. The authors highlighted how the lack of SLE diagnoses might reflect the clinicians’ inexperience and lack of familiarity with the disease’s presentation. This might partly be explained by the fact that throughout SSA there are limited training opportunities and work experience in paediatric rheumatology. Without adequate training, these diseases may be missed and therefore the prevalence under-estimated. Studies in the UK have shown that paediatric musculoskeletal education is poor throughout medical training, with primary care clinicians and paediatric trainees often having low confidence in relation to and therefore not carrying out musculoskeletal assessment [40,41]. A study in Nigeria found that every child in the SLE cohort had been misdiagnosed at initial presentation. The authors found that 36.4% of their patients had been originally treated for malaria because of the similarities in presentation and the initial response to hydroxychloroquine [4,42]. The presence of fever with myalgia is readily assumed to represent infection, while subtle rashes on dark skin often go unnoticed. If blood tests are undertaken, lymphopenia and anaemia may be wrongly attributed to chronic viral infection. The difficult presentation of SLE is not the only barrier to diagnosis because more specific investigations for SLE such as antinuclear antibody tests are often relatively expensive and not always available [43]. In adolescents, proteinuria in urinalysis often offers a clue to early SLE and should perhaps be considered a standard investigation for all patients presenting with multi-system features.

Although there is difficulty in diagnosing rheumatic disease, the low rates in this study might reflect its true rarity, especially in an inpatient setting, and are consistent with the low rates reported worldwide. In HIC, JIA and SLE were reported to have prevalences of 3.8–400/100,000 and 3.3–8.8/100,000, respectively [44,45]. Although rare, epidemiological research related to rheumatic disease should not be neglected because, if undiagnosed or poorly managed, JIA and SLE can lead to severe disability and in some cases can be fatal [46]. Previous studies have estimated quite striking figures for the prevalence of JIA and SLE in East Africa, which further supports the need for robust prevalence studies to accurately describe and address the health burden secondary to rheumatic disease. East Africa was estimated to have 9% of all global cases of SLE and JIA—18,779 and 187,787, respectively [47].

With the suggestion that the true prevalence of rheumatic disease is much greater than previously thought, coupled with the pressure already on health services owing to fractures and infections, the need for future studies to accurately assess and address the burden secondary to paediatric MSD is vital. Although the diagnosis and management of MSD in low-resource countries poses unique challenges, many initiatives have been suggested to improve outcome. The Paediatric Global Musculoskeletal Task Force is leading the way with three primary goals [48]. (i) To raise awareness: Awareness by clinicians can be improved at every stage, with universal changes in curricula which prioritise MSD education and assessment training [41]. In relation to rheumatic diseases, increasing patient education through resources such as information leaflets will help minimise delays in presentation owing to caregivers’ lack of disease awareness and fear of exclusion [49]. (ii) Identify and promote the right care: It is estimated that in SSA there are ten paediatric rheumatologists for approximately 550 million children [50]. To address this and the inadequacies in service provision, facilities and access to medication, resources and initiatives need to focus on paediatric non-communicable diseases [50,51]. Previously successful initiatives which have been cost-effective include telemedicine and the use of webinars to disseminate knowledge and experience. The Paediatric Society of the African League Against Rheumatism has run several successful webinar series across Africa [52]. Global collaborations have also been beneficial: the UWEZO project, a collaboration between Kenyan, British and Swedish rheumatologists, patients and researchers, has helped to train over 500 physicians throughout Kenya [53]. (iii) To promote healthy joints and bones: This can be partly achieved through changes in public health policy and campaigns advocating bone health [48].

This study was designed to be as accurate and reliable as possible, but it is not without limitations and bias. The findings must be interpreted in the context of these limitations. As with other retrospective studies, the accuracy of the results relies on the admitting clinician’s ability to correctly diagnose and record participant information. Because only the notes of children with an initial diagnosis of MSD were reviewed, those in whom the diagnosis was missed were not included. Previous studies have shown how during clerking paediatric musculoskeletal assessment is often poor and incomplete [40]. It must also be noted that only one of the ten recorded cases of septic arthritis had a positive culture, although all the cases presented like septic arthritis, and responded to treatment for septic arthritis. Without a positive culture, some of these cases might have been reactive or inflammatory arthritis instead. The absence of some data owing to missing notes also hinders the ability to draw reliable conclusions, but the demographic data for those whose overall data were missing is similar to the overall cohort. As those with missing data were excluded, the figures will be under-estimates. The study only reviewed data for inpatients aged 5–18 years. By excluding children under 5, the incidence of osteo-articular infections and fractures secondary to NAI may have been under-estimated as children in this age group are most at risk [29,35]. Children with mild disease not requiring an inpatient bed were not part of the study. This is likely to have a considerable effect on the number of rheumatic disease cases in the cohort. With the initial symptoms often being mild, these children can be managed in the primary care system or as an outpatient and seldom need admission to hospital. Children from the most deprived families may also have been missed, as out-of-pocket payments fall hardest on them and act as a barrier to their children being admitted to hospital [54]. Because the sample size was small with potential selection bias, the ability to extrapolate the findings and generalise the results to the wider population may be limited. However, this is the first publication of its kind from Tanzania and highlights the need for further studies in future, potentially looking at longer periods of data collection.

To conclude, this study describes the broad spectrum of paediatric MSD and highlights the great burden it imposes on inpatient services in Tanzania. Building on this study, larger, more comprehensive epidemiological studies should be undertaken in community and hospital settings. Robust prevalence studies would ensure inclusion of a range of presentations and severity of symptoms. Gaining a more accurate understanding of the spectrum, burden and factors which contribute to paediatric MSD can provide the information required for appropriate and effective resource and service provision in countries with limited financial resources. These studies would guide the development of appropriate public education as well as health and government policies which could ultimately have a significant impact on reducing the burden of paediatric MSD.

To the best of our knowledge, this is the first study in Tanzania to provide a snapshot of the spectrum and burden of paediatric MSD in an inpatient setting, an area of paediatric medicine that has often been overlooked. Although the cohort was small, the study identified a sizeable burden of paediatric MSD with a wide spectrum of conditions. Fractures followed by osteo-articular infections caused the greatest proportion of disease while rheumatic diseases were uncommon. This study supports previous literature which suggests that much of the burden of paediatric MSD in LMIC is secondary to preventable injury and infection.

## References

[1] Briggs AM, Cross MJ, Hoy DG, et al. Musculoskeletal health conditions represent a global threat to healthy aging: a report for the 2015 world health organization world report on ageing and health. Gerontologist. 2016;56:S243–S255. 26994264 10.1093/geront/gnw002

[2] Woolf AD, Pfleger B. Burden of major musculoskeletal conditions. Bull WHO. 2003;81:646–656. 14710506 PMC2572542

[3] Sebbag E, Felten R, Sagez F, et al. The world-wide burden of musculoskeletal diseases: a systematic analysis of the world health organization burden of diseases database. Ann Rheum Dis. 2019;78:844–848. 30987966 10.1136/annrheumdis-2019-215142

[4] Migowa A, Colmegna I, Hitchon C, et al. The spectrum of rheumatic in-patient diagnoses at a pediatric hospital in Kenya. Pediatr Rheumatol Online J. 2017;15:4–5. 28088248 10.1186/s12969-016-0131-3 PMC5237484

[5] Alves K, Penny N, Kobusingye O, et al. Paediatric musculoskeletal disease in Kumi District, Uganda: a cross-sectional survey. Int Orthop. 2018;42:1967–1973. 29610937 10.1007/s00264-018-3915-x PMC6469985

[6] Carter EW, Austin D, Trainor AA. Predictors of postschool employment outcomes for young adults with severe disabilities. J Disabil Policy Stud. 2012;23:50–63.

[7] Roux CH, Guillemin F, Boini S, et al. Impact of musculoskeletal disorders on quality of life: an inception cohort study. Ann Rheum Dis. 2005;64:606–611. 15576417 10.1136/ard.2004.020784 PMC1755431

[8] Mizunoya S, Mitra S, Yamasaki I. Towards inclusive education: the impact of disability on school attendance in developing countries. 2016. Accessed 01 July 2021. Available from: https://www.unicef-irc.org/publications/pdf/IWP3%20-%20Towards%20Inclusive%20Education.pdf

[9] Zajacova A, Lawrence EM. The relationship between education and health: reducing disparities through a contextual approach. Annu Rev Public Health. 2018;39:273–289. 29328865 10.1146/annurev-publhealth-031816-044628 PMC5880718

[10] Woolf AD, Brooks P, Akesson K, et al. Prevention of musculoskeletal conditions in the developing world. Best Pract Res Clin Rheumatol. 2008;22:759–772. 18783749 10.1016/j.berh.2008.07.003

[11] World Health Organization. ICD-10: international statistical classification of diseases and related health problems: tenth revision. Geneva: WHO; 2004. Accessed 15 June 2021. Available from: https://apps.who.int/iris/handle/10665/42980

[12] Rai S, Meng C, Wang X, et al. Gluteal muscle contracture: diagnosis and management options. SICOT J. 2017;3:1–2. 28059055 10.1051/sicotj/2016036 PMC5217396

[13] Alves K, Katz JN, Sabatini CS. Gluteal fibrosis and its surgical treatment. J Bone Joint Surg Am. 2019;101:361–368. 30801376 10.2106/JBJS.17.01670 PMC6738551

[14] World Health Organization. World malaria report 2019. Geneva: WHO; 2019. Accessed 20 June 2021. Available from: https://apps.who.int/iris/handle/10665/330011

[15] Alves K, Penny N, Ekure J, et al. Burden of gluteal fibrosis and post-injection paralysis in the children of Kumi District in Uganda. BMC Musculoskelet Disord. 2018;19:343. 30249239 10.1186/s12891-018-2254-9 PMC6154889

[16] Kiser MM, Samuel JC, McLean SE, et al. Epidemiology of pediatric injury in Malawi: burden of disease and implications for prevention. Int J Surg. 2012;10:611–617. 23142508 10.1016/j.ijsu.2012.10.004

[17] Hedström EM, Svensson O, Bergström U, et al. Epidemiology of fractures in children and adolescents. Acta Orthop Scand Suppl. 2010; 81: 148–153. 10.3109/17453671003628780 PMC2856220 20175744

[18] Valerio G, Gallè F, Mancusi C, et al. Pattern of fractures across pediatric age groups: analysis of individual and lifestyle factors. BMC Public Health. 2010;10:656–657. 21034509 10.1186/1471-2458-10-656 PMC2987399

[19] Morrongiello BA. Caregiver supervision and child-injury risk: i. Issues in defining and measuring supervision; II. Findings and directions for future research. J Pediatr Psychol. 2005;30:536–552. 16166243 10.1093/jpepsy/jsi041

[20] Deakin DE, Crosby JM, Moran CG, et al. Childhood fractures requiring inpatient management. Injury. 2007;38:1241–1246. 17888917 10.1016/j.injury.2007.05.023

[21] Parkes RJ, Parkes G, James K. A systematic review of cost-effectiveness, comparing traction to intramedullary nailing of femoral shaft fractures, in the less economically developed context. BMJ Glob Health. 2017;2:e000313. 10.1136/bmjgh-2017-000313 PMC5623315 29018580

[22] Ariës MJ, Joosten H, Wegdam HH, et al. Fracture treatment by bonesetters in central Ghana: patients explain their choices and experiences. Trop Med Int Health. 2007;12:564–574. 17445148 10.1111/j.1365-3156.2007.01822.x

[23] Stanifer JW, Patel UD, Karia F, et al. The determinants of traditional medicine use in Northern Tanzania: a mixed-methods study. PLoS One. 2015;10:e0122638–e. 25848762 10.1371/journal.pone.0122638 PMC4388565

[24] Ngunde PJ, Akongnwi ACN, Mefire CA, et al. Prevalence and pattern of lower extremity injuries due to road traffic crashes in Fako Division, Cameroon. Pan Afr Med J. 2019;32:53–54. 31143358 10.11604/pamj.2019.32.53.17514 PMC6522147

[25] Heydari S, Hickford A, McIlroy R, et al. Road safety in low-income countries: state of knowledge and future directions. Sustainability. 2019;11:6249.

[26] Stevenson M, Thompson J. On the road to prevention: road injury and health promotion. Health Promot J Austr. 2014;25:4–7. 24739772 10.1071/HE13075

[27] Moody G, Cannings-John R, Hood K, et al. Establishing the international prevalence of self-reported child maltreatment: a systematic review by maltreatment type and gender. BMC Public Health. 2018;18:1164–1165. 30305071 10.1186/s12889-018-6044-y PMC6180456

[28] Karmazyn B, Lewis ME, Jennings SG, et al. The prevalence of uncommon fractures on skeletal surveys performed to evaluate for suspected abuse in 930 children: should practice guidelines change? Am J Roentgenol. 2011;197:W159–W163. 21700979 10.2214/AJR.10.5733

[29] The Royal College of Paediatrics and Child Health. Child protection evidence – systematic review on fractures, Cardiff University, 2020. Accessed 21 January 2022. Available from: https://www.rcpch.ac.uk/sites/default/files/2020-10/Chapter%20Fractures_Update_280920.pdf

[30] African Partnership to End Violence Against Children A. Violence against children in Africa: a Report on Progress and Challenges. African Child Policy Forum; 2020. Accessed 21 January 2022. Available from: https://violenceagainstchildren.un.org/sites/violenceagainstchildren.un.org/files/2021/violence_against_children_in_africa_a_report_on_progress_and_challenges.pdf.

[31] Dartnell J, Ramachandran M, Katchburian M. Haematogenous acute and subacute paediatric osteomyelitis: a systematic review of the literature. J Bone Joint Surg Br. 2012;94:584–595. 22529075 10.1302/0301-620X.94B5.28523

[32] Stanley CM, Rutherford GW, Morshed S, et al. Estimating the healthcare burden of osteomyelitis in Uganda. Trans R Soc Trop Med Hyg. 2010;104:139–142. 19709706 10.1016/j.trstmh.2009.05.014

[33] Mantero E, Carbone M, Calevo MG, et al. Diagnosis and treatment of pediatric chronic osteomyelitis in developing countries: prospective study of 96 patients treated in Kenya. Musculoskelet Surg. 2011;95:13–18. 21373913 10.1007/s12306-011-0104-0

[34] Jones HW, Beckles VL, Akinola B, et al. Chronic haematogenous osteomyelitis in children: an unsolved problem. J Bone Joint Surg Br. 2011;93:1005–1010. 21768620 10.1302/0301-620X.93B8.25951

[35] Iliadis AD, Ramachandran M. Paediatric bone and joint infection. EFORT Open Rev. 2017;2:7–12. 28607765 10.1302/2058-5241.2.160027 PMC5444236

[36] Millet A, Hullo E, Armari Alla C, et al. Sickle cell disease and invasive osteoarticular Salmonella infections. Arch Pediatr. 2012;19:267–270. 22261260 10.1016/j.arcped.2011.12.012

[37] Almeida A, Roberts I. Bone involvement in sickle cell disease. Br J Haematol. 2005;129:482–490. 15877730 10.1111/j.1365-2141.2005.05476.x

[38] Dunn RN, Ben Husien M. Spinal tuberculosis: review of current management. Bone Joint J. 2018;100-b:425–431. 29629596 10.1302/0301-620X.100B4.BJJ-2017-1040.R1

[39] Spiegel DA, Nduaguba A, Cherian MN, et al. Deficiencies in the availability of essential musculoskeletal surgical services at 883 health facilities in 24 low- and lower-middle-income countries. World J Surg. 2015;39:1421–1432. 25663008 10.1007/s00268-015-2971-2

[40] Myers A, McDonagh JE, Gupta K, et al. More ‘cries from the joints’: assessment of the musculoskeletal system is poorly documented in routine paediatric clerking. Rheumatology (Oxford). 2004;43:1045–1049. 15187245 10.1093/rheumatology/keh245

[41] Jandial S, Myers A, Wise E, et al. Doctors likely to encounter children with musculoskeletal complaints have low confidence in their clinical skills. J Pediatr. 2009;154:267–271. 18823907 10.1016/j.jpeds.2008.08.013

[42] Olowu W. Childhood-onset systemic lupus erythematosus. J Natl Med Assoc. 2007;99:777–784. 17668644 PMC2574347

[43] World Health Organization Alliance for Health Policy Systems Research. Primary health care systems (primasys): comprehensive case study from United Republic of Tanzania. Geneva: WHO; 2017. Accessed 21 January 2022. Available from: https://apps.who.int/iris/handle/10665/341165

[44] Manners PJ, Bower C. Worldwide prevalence of juvenile arthritis why does it vary so much? J Rheumatol. 2002;29:1520–1530. 12136914

[45] Thierry S, Fautrel B, Lemelle I, et al. Prevalence and incidence of juvenile idiopathic arthritis: a systematic review. Joint Bone Spine. 2014;81:112–117. 24210707 10.1016/j.jbspin.2013.09.003

[46] Tan J, Fun H, Arkachaisri T. Paediatrics rheumatology clinic population in Singapore: the KKH experience. Proc Singapore Healthc. 2012;21:265–271.

[47] Dave M, Rankin J, Pearce M, et al. Global prevalence estimates of three chronic musculoskeletal conditions: club foot, juvenile idiopathic arthritis and juvenile systemic lupus erythematosus. Pediatr Rheumatol Online J. 2020;18:49–50. 32532304 10.1186/s12969-020-00443-8 PMC7291758

[48] Foster HE, Scott C, Tiderius CJ, et al. The paediatric global musculoskeletal task force - ‘towards better MSK health for all’. Pediatr Rheumatol Online J. 2020;18:60. 32664961 10.1186/s12969-020-00451-8 PMC7359433

[49] Lewandowski LB, Schanberg LE, Thielman N, et al. Severe disease presentation and poor outcomes among pediatric systemic lupus erythematosus patients in South Africa. Lupus. 2017;26:186–194. 27488473 10.1177/0961203316660625 PMC5290292

[50] Scott C, Chan M, Slamang W, et al. Juvenile arthritis management in less resourced countries (JAMLess): consensus recommendations from the cradle of humankind. Clin Rheumatol. 2019;38:563–575. 30267356 10.1007/s10067-018-4304-y

[51] Henrickson M. Policy challenges for the pediatric rheumatology workforce: part II. Health care system delivery and workforce supply. Pediatr Rheumatol Online J. 2011;9:24. 21843335 10.1186/1546-0096-9-23 PMC3173344

[52] Migowa AN, Hadef D, Hamdi W, et al. Pediatric rheumatology in Africa: thriving amidst challenges. Pediatr Rheumatol Online J. 2021;19:69–70. 33962643 10.1186/s12969-021-00557-7 PMC8103667

[53] Erwin J, Woolf A, Oyoo O, et al. The UWEZO project-musculoskeletal health training in Kenya. Clin Rheumatol. 2016;35:433–440. 25596014 10.1007/s10067-015-2863-8

[54] Schellenberg JA, Victora CG, Mushi A, et al. Inequities among the very poor: health care for children in rural southern Tanzania. Lancet. 2003;361:561–566. 12598141 10.1016/S0140-6736(03)12515-9

